# A Novel Long-term, Multi-Channel and Non-invasive Electrophysiology Platform for Zebrafish

**DOI:** 10.1038/srep28248

**Published:** 2016-06-16

**Authors:** SoonGweon Hong, Philip Lee, Scott C. Baraban, Luke P. Lee

**Affiliations:** 1 Department of Bioengineering Engineering, University of California, Berkeley, CA 94720, USA; 2 Berkeley Sensor and Actuator Center, University of California, Berkeley, CA 94720, USA; 3 Epilepsy Research Laboratory, Department of Neurological Surgery, University of California at San Francisco, USA, CA 94143, USA; 4 Eli and Edythe Broad Center of Regeneration Medicine and Stem Cell Research, University of California at San Francisco, USA, CA 94143, USA; 5 Department of Electrical Engineering and Computer Sciences, University of California, Berkeley, CA 94720, USA; 6 Biophysics Graduate Program, University of California, Berkeley, CA 94720, USA

## Abstract

Zebrafish are a popular vertebrate model for human neurological disorders and drug discovery. Although fecundity, breeding convenience, genetic homology and optical transparency have been key advantages, laborious and invasive procedures are required for electrophysiological studies. Using an electrode-integrated microfluidic system, here we demonstrate a novel multichannel electrophysiology unit to record multiple zebrafish. This platform allows spontaneous alignment of zebrafish and maintains, over days, close contact between head and multiple surface electrodes, enabling non-invasive long-term electroencephalographic recording. First, we demonstrate that electrographic seizure events, induced by pentylenetetrazole, can be reliably distinguished from eye or tail movement artifacts, and quantifiably identified with our unique algorithm. Second, we show long-term monitoring during epileptogenic progression in a *scn1lab* mutant recapitulating human Dravet syndrome. Third, we provide an example of cross-over pharmacology antiepileptic drug testing. Such promising features of this integrated microfluidic platform will greatly facilitate high-throughput drug screening and electrophysiological characterization of epileptic zebrafish.

High-resolution neural recording of brain activity is an essential tool in basic neuroscience and remains a “gold standard” for disease diagnosis in epilepsy. Clinically, the epilepsies are characterized by abnormal excessive neuronal discharge within the central nervous system and are commonly identified using an electroencephalogram (EEG)^1^. In animal models, EEG detection and monitoring of spontaneous recurrent seizure events is also crucial to the accurate identification of epileptic phenotypes and evaluation of therapies^2^,^3^. Surface screws or penetrating metal microelectrode arrays commonly used for EEG in rodents require surgical implantation and may lead to inflammation- or tissue damage-evoked electrographic seizure events. An alternative experimental vertebrate recently shown to be useful in modeling acute or genetic epilepsies (i.e., zebrafish) can be monitored in an agarose-embedded preparation using a glass microelectrode inserted into the brain^4^,^5^,^6^,^7^. Small size, optical transparency and fecundity make zebrafish (*Danio rerio*) larva an ideal organism for *in vivo* studies of fundamental neurobiological processes^8^,^9^,^10^ as well as large-scale screening of chemical libraries in disorder-specific epileptic zebrafish^6^,^11^. The high resolution neural recordings obtained in these epileptic zebrafish exhibit electrical patterns that are fundamentally similar to those seen in rodents and humans^12^. Moreover, zebrafish offer tremendous advantages over rodents for (i) rapid genetic manipulation and precision modeling of human epilepsies^13^ and (ii) high-throughput drug discovery^14^. Although its optical transparency permits brain-wide imaging of neuronal activity using genetically encoded calcium indicators^15^,^16^ or direct placement of field electrodes to record from selected brain structures^17^, both approaches require immobilization in agarose and are relatively short-term in nature e.g., minutes to hours. As of yet, long-term non-invasive EEG monitoring using multiple electrode arrays on more than one fish simultaneously has not been possible.

Advances in microfabrication techniques have led to the design of microfluidic devices for the manipulation of small organisms, including zebrafish larvae^18^,^19^,^20^,^21^. A microfluidic chip designed to allow automatic trapping, positioning and orientation of zebrafish larvae was used to obtain calcium imaging data during various drug manipulations^20^. Candelier *et al*. designed a similar microfluidic device, incorporated with solution delivery channels, to precisely deliver chemical stimuli during optical imaging of neural activity and tail movement^21^. Rapid *in vivo* imaging of zebrafish larvae has also been achieved using a fluidic device that briefly restrains larva in a capillary tube in the field of view of a confocal or widefield microscope objective^22^. Each of these approaches were designed to isolate a single zebrafish, and were not described as having the capacity to study multiple zebrafish simultaneously or for prolonged monitoring periods e.g., hours to days. Here we describe an integrated Zebrafish Analysis Platform (iZAP) for long-term non-invasive high-throughput multichannel electrophysiological monitoring. In contrast to earlier zebrafish microfluidic platforms^19^,^20^,^21^,^23^,^24^, iZAP system can restrain several freely swimming zebrafish larvae autonomously and simultaneously underneath multiple integrated microelectrodes within the microfluidic chamber array, simply by depositing larvae into a microfluidic inlet pool; zebrafish restrained in the microfluidic device are stable for several days while allowing solution exchanges. Together with integrated custom-built multichannel head-stage amplifiers, surface microelectrodes contacting zebrafish heads continuously monitor multiple EEG signals from multiple zebrafish with high sensitivity and low electrical noise. Such long-term non-invasive electrical readout facilitates reliable, robust, statistical analysis of zebrafish electrophysiology and significantly reduces the time and cost required to validate antiepileptic drugs.

## Results

### Design of integrated Zebrafish Analysis Platform (iZAP)

**Figure 1:**
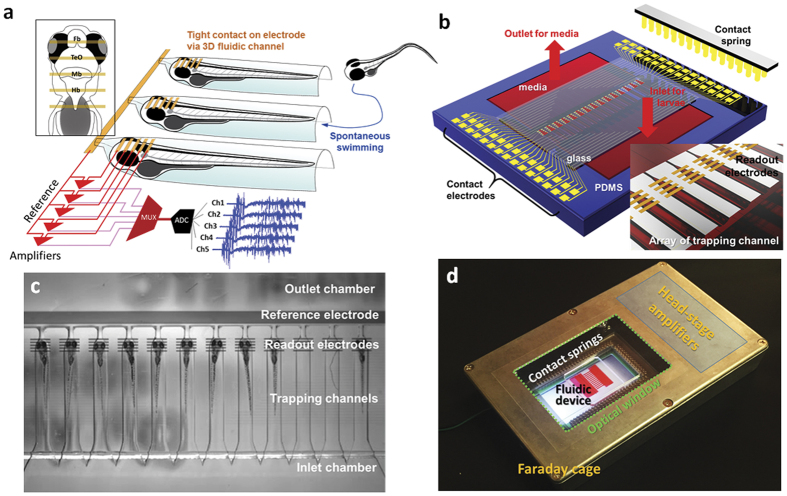
Integrated zebrafish analysis platform (iZAP) for high-throughput long-term electrophysiological monitoring. **(a)** Scheme of the iZAP design concept. The inset shows the relative position of surface electrodes to zebrafish brain (Fb, forebrain; TeO, optic tectum; Mb, midbrain; Hb, hindbrain) and a multichannel amplifier (MUX, multiplexer; ADC, analog-to-digital converter). **(b)** An illustration of the integrated microfluidic platform. **(c)** Picture of zebrafish array in the iZAP microfluidic unit. **(d)** A picture of iZAP system inside an optical-windowed Faraday cage. The red fluid in the fluidic device represents zebrafish media.

The iZAP system consists of three primary components: (i) microfluidic unit, (ii) multielectrode array, and (iii) integrated electronic unit with multichannel amplifiers. The microfluidic unit is designed as an open chamber with a large-volume inlet pool (22 mm height × 8 mm width × 0.8 mm depth) where loaded zebrafish larvae are allowed to freely swim (Fig. 1a,b). Twelve parallel channels (6 mm length × 0.8 mm width × 0.8 mm depth) are shaped as half-cylinders with one end open to the inlet chamber and the opposite end gradually tapered as a half-cone. A small microfluidic channel (0.5 mm length × 0.1 mm width × 0.1 mm depth) connects the cone-shaped end with a large-volume outlet chamber (details in Fig. S1). The length of an individual channel is determined to accommodate a single zebrafish larvae aged 3 to 7 days post-fertilization (dpf; length 3.6 to 4.2 mm). Zebrafish are deposited in the inlet chamber and are free in the inlet pool to spontaneously swim into a single channel where they become restrained, dorsal side up, at the head due to the tapered half-cylindrical channel shape; fish were very rarely observed to escape from the channel once restrained. Outlet and inlet chambers are designed to allow direct pipetting or micro-perfusion of media.

Integrated microelectrodes with an insulating layer were fabricated onto a transparent polyethylene terephthalate (PET) substrate and applied to measure field potentials spanning the anterior-to-posterior aspect of the zebrafish forebrain, optic tectum, midbrain and hindbrain. Surface microelectrodes are patterned with transparent indium tin oxide (ITO). Electrodes are coated with a thin 10 nm platinum layer for enhanced electrical stability and direct visualization of zebrafish under the readout electrodes. Dielectric passivation on the patterned electrode is applied to protect surface electrodes from an electrolyte as 10-μm thick SU8. Surface electrode configuration consists of a set of five contact electrodes separated by 200 μm and positioned to tightly contact the head of a restrained zebrafish larva. A large-area reference electrode is positioned on the outlet chamber to circumvent any electrical disturbances. The readout electrodes are connected to contact pads distributed along the edges of the 2” × 3” PET substrate, as matched to the array of spring contacts of the electronic unit (Fig. 1b). After the fabrication of the multielectrodes, two cutouts for the inlet and the outlet were made using a laser cutter (VersaLaser VL-200), and the microfluidic unit was bonded on the substrate with an oxygen plasma treatment (58 W RF power, 10 sccm O_2_, PETS Reactive Ion Echer).

An in-house built circuit board (160 mm × 90 mm) is integrated with two 32-channel head-stage amplifiers and an array of spring contacts with an inner cut for optical visualization. Head-stage amplifiers are designed to be close to readout positions to minimize electrical noise, and allow the total integrated system to be placed within a small-size electrical shielding cage (185 mm length × 120 mm width × 35 mm height; Fig. 1d) on a conventional stereoscope for simultaneous video monitoring. Connected to an external ADC converter and computer running custom software, all 60 electrical readouts from up to 12 zebrafish are simultaneously recorded at 1 kHz. To characterize the performance of the microfluidic unit, we loaded nine to ten zebrafish to the inlet chamber and let them freely swim into individual chambers *c.a*. 30 min; experiments were performed on 8 independent clutches of zebrafish (Fig. 1c). After long-term electrographic monitoring or drug treatments described in more detail below, we successfully retrieved individual zebrafish by pipetting, and released them into petri dishes where they appeared healthy and could be raised, with appropriate care and feeding, to adulthood.

### Electrographic recording from an acute seizure model

**Figure 2:**
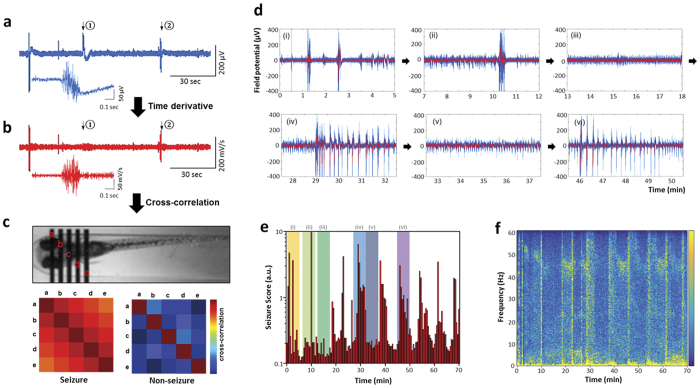
Electrographic seizure recording and seizure score algorithm. **(a)** An electroencephalograph recorded from a surface electrode contacted to a zebrafish head. A zoomed electrograph was shown for the instance noted as ②. **(b)** Corresponding time-derivative of electrograph in (**a**). A zoomed time-derivate was shown for the instance ②. **(c)** Cross-correlation between time-derivatives of spatially distributed five electrode signals. The seizure score is a summation of the off-diagonal numbers of the cross-correlation set and can effectively screen out non-seizure-like electrographic signals like ①. **(d)** Representative EEG episodes of a PTZ-induced epilepsy model showing ictal- and interictal-like bursts. The red line is averaged from nearby 10 data point attached as 1 kHz. **(e)** Seizure score vs. time plot for the PTZ-induced epilepsy model. Time spans for the representative EEG episodes are indicated as Roman numerals. **(f)** Corresponding EEG spectra vs. time.

To demonstrate the feasibility of monitoring multiple EEG signals from zebrafish restrained in microfluidic channels, we first performed acute electrophysiology studies using wild-type zebrafish. Bath application of a common convulsant agent, pentylenetetrazole (PTZ), validated to evoke seizures in zebrafish^5^ was used as a “proof-of-principle”. After restraining WT zebrafish larvae (5–7 dpf) as described above, we first monitored electrographic baseline activity from surface electrodes (5 per fish) for one hour. As expected^5^,^6^,^13^,^25^, all electrodes showed electrical signals less than 50 μV in amplitude and no evidence of abnormal burst discharge activity. 10 mm PTZ was added to the loading chamber and within minutes we observed abnormal electrical discharges. Starting around 10 min after PTZ exposure, multi-spike large amplitude ictal-like burst discharges (Fig. 2d, iii, v) and repeating interictal-like bursts with relatively small amplitudes (Fig. 2d iv, vi) were detected. These types of electrographic seizure events evolved to be more complex at approximately 30 min of PTZ exposure. Seizure episodes were observed in all PTZ exposed larvae (n = 30). Seizure activity peaked and waned in a periodic fashion at roughly 15 min intervals (Fig. 2e). These electrographic events are comparable, though smaller in amplitude, to those obtained by directly inserting a glass microelectrode into the zebrafish brain of agarose-embedded WT zebrafish (Fig. S2). To confirm that detected epileptiform electrographic episodes can be distinguished from zebrafish motion artifacts, we exposed a separate clutch of zebrafish to paralyzing agents (1 mg·ml^−1^ *α*-Bungarotoxin or 300 μM pancuronium bromide) prior to and during co-application of PTZ (Figs S3 and S7). Movement artifacts in the electrographic recordings were significantly reduced in the presence of *α*-Bungarotoxin (66.9 ± 25.6%; n = 9) or pancuronium (62.3 ± 22%; n = 7). During co-application of PTZ with either *α*-Bungarotoxin or pancuronium, we recorded an identical series of electrographic seizure events (Fig. S3).

### Seizure scoring software

The electrophysiological characteristics of a seizure differ dramatically from baseline events. Detecting these electrographic events in real-time can be difficult and requires specialized algorithms. Here we developed a unique software algorithm to identify and measure putative seizure events observed in larval zebrafish. This algorithm is compatible with real-time measurement of big data sets (i.e., up to 1.8 × 10^8^ data points per second in the iZAP system) or *post hoc* off-line analyses. The algorithm is based on distinct features of the zebrafish electrographic seizure signals and measures (i) how abruptly the electrical signal changes from baseline and (ii) the extent of spatial correlation between the 5 surface electrodes. First, the software measures a time derivative from the recorded electrical potentials so that electrical signals associated with movements (described in the next section) are scored less than an abrupt complex seizure-event. For example, while the representative electrical signal shown in Fig. 2a contains similar-amplitude electrical responses from a movement artifact and a seizure event (noted as (i) and (ii) in the plot, respectively), the time derivative filters out low-frequency field changes as shown in Fig. 2b and only scores electrographic events with rapid complex changes. Second, cross-correlations among five different time derivatives from the five-electrode set are followed to quantify the similarity of electrical signal change (Fig. 2c). The following equation shows the mathematical expression for the algorithm.


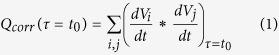


where 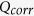 is the cross-correlation index, *t*_0_ is time of interest, *V*_*i*_ is a measured field potential from *i*^*th*^ surface electrode, and *i* and *j* range in 1 to 5. Then, a time integration of the cross-correlation index over a relatively long period (i.e., 30 sec in Fig. 2e) is applied so that brief motion artifacts or electrical noises can be additionally less scored in the seizure-scoring plot (see a representative example in Fig. S6). The continuous measurement of acute PTZ-evoked seizure events can be concisely expressed as a bar plot, where individual bar heights inform how abrupt electrical discharge happens and how often putative seizure episodes occur (thus, as an index of seizure extent) in a certain time period. The bar plot in Fig. 2e summarized from 70 min of continuous PTZ exposure also reveals the periodic clustering of seizure episodes in this model. Individual interictal-like spikes occurred periodically with a narrow band of frequency between 40 and 50 Hz (Fig. 2f), while the ictal spikes occurred as broad-band frequency emphasized in the low frequency region below 5 Hz.

### Detection of movement artifacts

**Figure 3:**
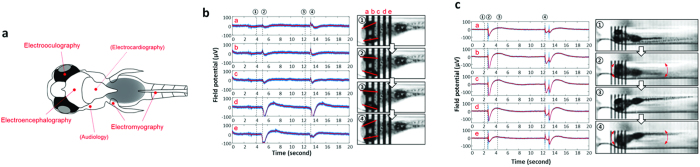
Distinguishing epileptic artifacts in iZAP. **(a)** Various electrophysiology modalities detectable in iZAP system. The current iZAP system can distinguish seizure-like EEG signals from **(b)** eye and **(c)** tail (i.e., electrooculography and electromyography). The corresponding videographs were taken as 30 frames per second.

Because the iZAP system utilizes non-invasive surface electrode recordings from restrained (but not agarose embedded) zebrafish larvae, other types of electrical activities can also be detected (Fig. 3a). Our preliminary comparison of the video during these electrical recordings highlights a few representative examples. First, the most prevalent electrographic non-seizure events relate to eye motion (Fig. 3b). Large-amplitude slow electrical signals from the five-electrode set were detected during a several-second frame synchronized with a single eye motion. Immediately after an eye motion, the highest amplitude of signal was detected from the 4^th^ electrode positioned around the hindbrain, while more rostral electrodes (i.e., the 1^st^ electrode) captured a 180° off-phase forgoing signal with smaller amplitudes. The set of electrical signals rapidly rose from the baseline and restored as a slow long-tailed decrease in 2 to 3 seconds, similarly to eye-associated artifacts in animal or human EEG^26^,^27^,^28^,^29^. Second, electrical signals could be detected in association with tail movements (Fig. 3c). In this case, a short-term electrical pulse preceded and slow long-tailed decrease followed from all the 5 electrodes synchronously. These two movement-associated electrical activities were mostly observed at a slow frequency of a few times per minute in our experiments with zebrafish larvae and were not commonly observed in paralyzed zebrafish larvae (see Figs S3 and S7).

### Long-term measurement using a genetic epilepsy model

**Figure 4:**
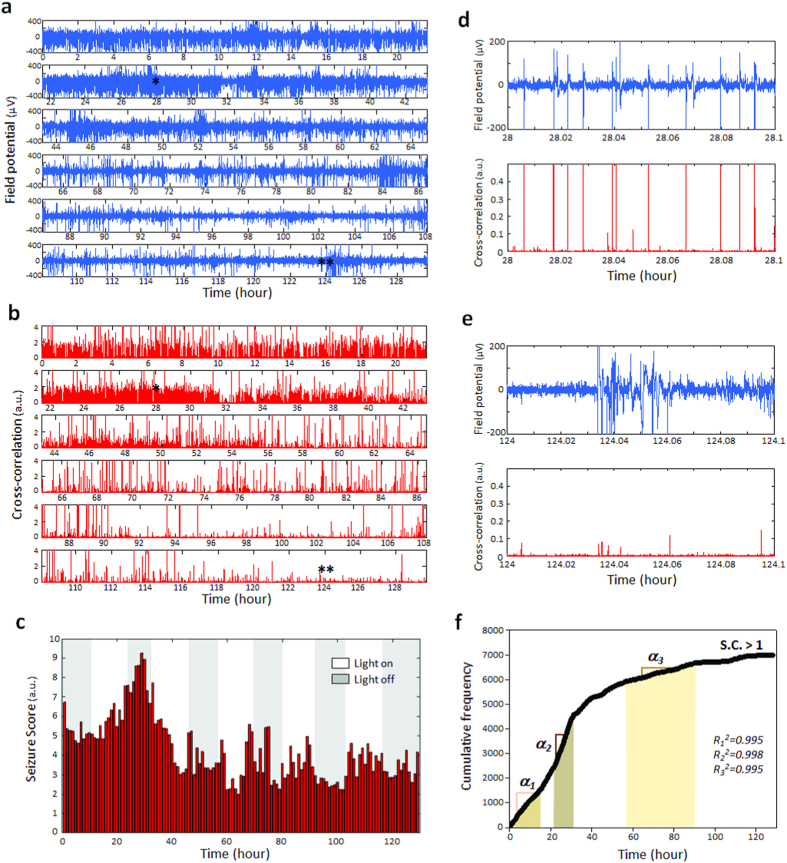
Long-term EEG monitoring capability of the iZAP system. Scn1Lab mutants of a chronic epilepsy model were restrained in the iZAP system between 3 to 8 dpf and continuously monitored with the surface electrodes. **(a,b)** Representative electrograph and corresponding cross-correlation plot of 130 hours continuous monitoring. **(c)** 1-hour interval seizure score bar plot overlapped with circadian light cycle applied. **(d,e)** Zoom-ins of the electrograph and the time-derivative. At the maximal seizure activity at 28 hour, high-amplitude field potentials resulted in high-magnitude seizure scores while at the later stage like 8 dpf, high-amplitude field potentials were not related to seizure-like bursts. **(f)** Cumulative frequency plot for seizure-like bursts over a threshold of seizure score (seizure score >1). Three linear regions were found as indicated with α_1_, α_2_ and α_3_. Each corresponding R^2^ is indicated in the plot.

The capacity for long-term non-invasive electrophysiological monitoring can be a potential basis for phenotypic analysis of genetically modified zebrafish or high-throughput pharmaceutical applications. Available invasive electrographic monitoring methods using agarose immobilization and penetrating microelectrodes are only viable for several hours and media wash-out is difficult with agarose embedded larvae. To investigate the feasibility of using the iZAP system for chronic recordings we used a *scn1* Dravet syndrome (DS) mutant zebrafish previously shown to exhibit electrographic seizure activity between 3 and 7 dpf^6^,^25^. For this experiment, *scn1Lab* mutants screened from larval clutches based on pigmentation were loaded to the iZAP at 3 dpf and continuously monitored until 8 dpf. During this recording period, restrained zebrafish remained in the channels and media was changed at 12 hour intervals with less than 2-min recording interruption. The nearly continuous 130-hour electrographic data was recorded and Fig. 4a presents a representative recording from the *scn1Lab* mutant. The cross-correlation plot in Fig. 4b indicates that epileptiform electrographic events are maximal at 3–5 dpf and show an age-dependent light-dark sensitivity with increased seizure activity during the dark periods at 3–6 dpf. With progressing age, detected electrographic seizure events became less prominent and less sensitive to light-dark cycles. Summarized as a one-hour-interval bar plot overlapped with the circadian light cycle (Fig. 4c), the maximal seizure score positions peaks around 4 dpf. At the conclusion of the 130-hr electrical recording period, we transferred *scn1Lab* zebrafish larvae to a media bath and compared locomotion with age-matched siblings maintained in a petri dish containing embryo media (Supplementary movie 1). No obvious differences in locomotion and appearance were observed. Our tracing of the sequence of seizure-like bursts found that some electrographic events with a high amplitude of field potential did not relate to seizure-like episodes in the later stage (i.e., 7–8 dpf) of the monitoring (Fig. 4d vs. 4e) and that a three-stage frequency trend of seizure bursting could be recognized as shown in Fig. 4f.

To further confirm the identity of abnormal seizure-like bursts in the electrographs, we compared electrical activities of the 5-dpf *scn1Lab mutants* with and without a paralyzing agent, pancuronium bromide (Fig. S8). In both types of recording no obvious variation in the electrographic seizure monitoring was observed. We failed to observe electrical events classified as abnormal burst discharge (i.e., seizure) in any age-matched control (Fig. S8), as reported previously^30^.

### Drug screening using the iZAP system

**Figure 5:**
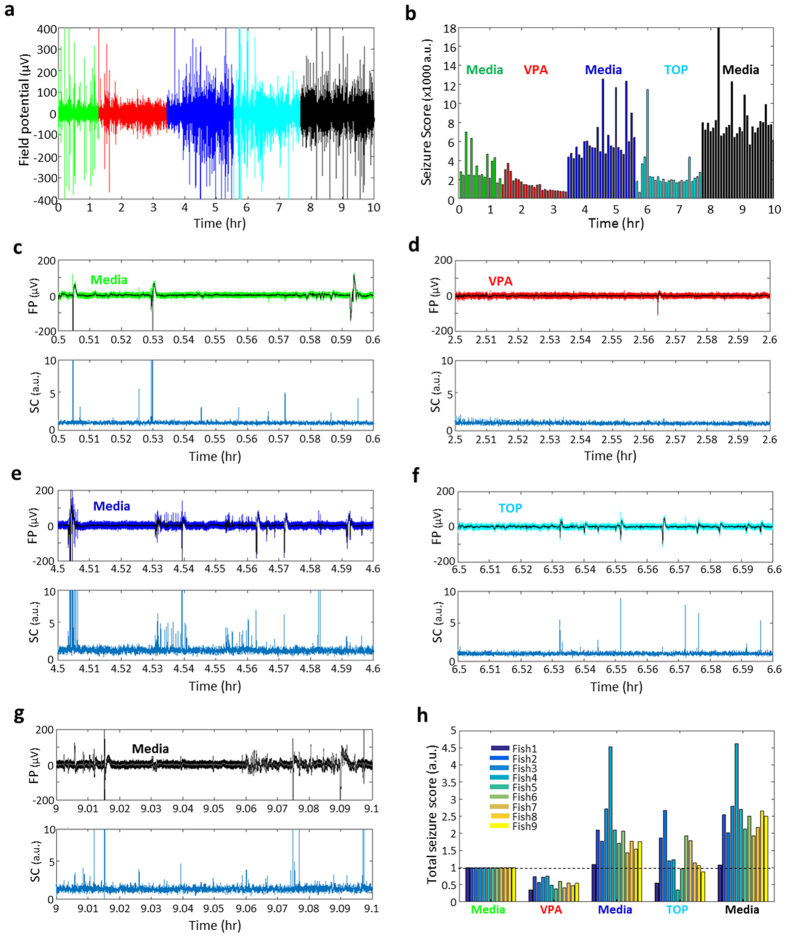
Drug screening demonstration of iZAP system. **(a,b)** A representative EEG signals and corresponding 5-min interval seizure score bar plot for VPA and TOP crossover test. **(c–g)** Electrograph and corresponding cross-correlation plot for each stage of the crossover test. In the label, FP is for field potential, and CC is for cross-correlation. **(h)** Drug efficacy plot based on total seizure score from 9 *scn1Lab* mutants as baseline-normalized.

Phenotype-based screening in zebrafish is emerging as a critical component of the drug discovery process^30^. Locomotion based assays allow for high-throughput testing in zebrafish epilepsy models^31^,^32^ but ultimate identification of an antiepileptic drug requires electrophysiological testing. As existing electrophysiology protocols require the agarose embedding of a single larvae and slow diffusion of test compounds into the agar, a system for higher throughput and direct bath exposure to the test compound would greatly improve this process. Here we used *scn1Lab* mutants with chronic spontaneous seizures (see Fig. 4) to provide a “proof-of-principle” demonstration of the use of a genetic zebrafish epilepsy model in a double-blind cross-over testing protocol^33^,^34^,^35^ (Fig. 5). Two antiepileptic drugs showing some clinical efficacy in DS patients^36^–valproic acid (VPA) and topiramate (TOP)–were selected, prepared and coded by an investigator blind to the iZAP experiment. As described above, *scn1Lab* mutants (6 dpf) were sorted by pigmentation and restrained in individual channels for EEG monitoring. Baseline measurement of spontaneous seizure activity was collected for 1.5 h (Fig. 5a). Coded media containing VPA (or TOP) was washed on for 2 hr, followed by a 2 hr embryo media wash-out period, and crossover drug application with VPA (or TOP) for another 2 hr, followed by a final 2 hr embryo media wash-out (Fig. 5a,f). Each treatment or wash-out period included a static bath exposure and media exchanges without any disturbance of the larvae. EEG activity was simultaneously monitored on all 5 channels of the multielectrode array for 9 *scn1Lab* mutant zebrafish and sample recordings (top) with corresponding seizure identification analysis (bottom) are shown for media conditions (Fig. 5c,e,g), VPA (Fig. 5d) and TOP (Fig. 5f). In the VPA-TOP treatment, all 9 mutant larvae responded positively in VPA treatment (i.e., decrease of seizure score), and 7 of 9 larvae positively responded during TOP treatment when compared to each previous baseline (Fig. 5h). A similar experiment with TOP followed by VPA was also performed on separate batch of *scn1Lab* mutant zebrafish (see Fig. S5).

## Discussion

Our results demonstrate that an iZAP system can achieve high-throughput, non-invasive, and long-term electrophysiological monitoring in simple vertebrate epilepsy models. In addition to the promising electrical recording capability, the most encouraging characteristic of the iZAP system for applications is the user-friendly long-term operation. Previous electrophysiological recordings in zebrafish required laborious processes such as agarose immobilization and delicate placement of micro-needle electrodes under skin, which are major limitations on data throughput (e.g., single fish with a single electrode per a single-time operation) and long-term survival of the larva. Using the iZAP system, spontaneous trapping by swimming from the inlet pool to restraining channels of zebrafish aligned to recording electrodes can be achieved with simple transfer of multiple zebrafish into the loading chamber, and desired individual zebrafish can be retracted back to inlet chamber by pipetting for genotyping, further characterization, or raising to adulthood. We also demonstrate that additional physical restrictions (i.e., agarose or muscle relaxants) are not necessary to obtain high fidelity recordings, which further highlights the non-invasive nature of our iZAP system and limits any drug-drug interactions that may complicate pharmacology studies. Integrated with multiple microelectrodes and multiple microfluidic channels, the iZAP system is capable of simultaneously monitoring up to 12-zebrafish larvae and the electrical recordings obtained are comparable to those seen with more invasive single-zebrafish recording techniques^5^,^6^,^7^,^11^,^19^. Because zebrafish released from the iZAP system at the conclusion of a recording period are viable, this system is also compatible with large-scale phenotype-genotype characterization of new zebrafish mutants and the subsequent raising of larvae of interest. In addition, the convenient operation and low cost allows one to perform several sets of simultaneous experiments with multiple iZAP systems in a laboratory scale. With multiple electrodes per zebrafish one may capture various electrical episodes corresponding to electroencephalography, electromyography, electrooculography and audiology (Figs 1a, 2c and 3a). The overall design of this system holds an additionally available 68 electrodes for these purposes. As a consequence, our next generation of iZAP system will potentially provide a wide spectrum of electrophysiological characterization tools for various researches and pharmaceutical fields.

In addition to the user-friendly hardware configuration, our unique algorithm allows for efficient handling of large electrographic data sets. Based on essential characteristics of epileptic electrographic seizures, our algorithm reliably detects and scores putative seizure events. The algorithm quantifies electrographic similarity among 100-millisecond continuous data sets of the five 1 kHz reads, and thus the algorithm reduces the size of data ~150 × without losing sensitivity of the seizure scoring since poly-spike events usually occur in durations greater than 100 milliseconds. In our study, the 1-day data set size from 12-fish electrodes is about 10 gigabytes, but the 1-day events can be summarized with a smaller size than 70 megabytes as the seizure score and the 5-day seizure score is as small as 350 megabytes. In consideration of current general computing capacity, this level of data reduction offers an appealing feature for large-scale seizure investigations.

Our proof-of-concept validation with zebrafish larvae addresses some distinguishable features of zebrafish epilepsy especially in terms of the electrographic progression (or “epileptogenesis”) that evolves with acute convulsant exposure or chronic genetic epilepsy models. With prolonged PTZ exposure and continuous recording, we observed, for the first time, ictal-like electrographic bursts that occur in a random manner within the first 10 min of PTZ treatment, and with time, clustered repeating of ictal- and interictal-like bursts during the 1–2 hour monitoring period. Clustering of seizures has been reported in rodent models with kainic acid or pilocarpine using long-term video-EEG monitoring^37^,^38^,^39^ but not in zebrafish. In *scn1Lab* mutants recapitulating Dravet syndrome^6^, a progressive development of seizure-like activity was observed with age in the absence of paralyzing agent applications while maintaining an electrographic seizure event detection sensitivity similar to that recorded in paralyzed larvae (Fig. S7). Progressive increases in the frequency of spontaneous seizures have been detected in acquired rodent epilepsy models after an acute period of status epilepticus^39^,^40^,^41^,^42^ but have not been studied in genetic epilepsy models. When individual electrographic events were counted based on a cross-correlation threshold (normalized cross-correlation >1) in the cross-correlation plot (Fig. 4b)^39^, a linear region was found around the maximal seizure activity from the cumulative histogram (*α*_*2*_ region in Fig. 4f), where the seizure-like bursts were periodically chained with short-term interval of *c.a*. 30 sec (Fig. S4). Before reaching the maximal seizure activities, a periodic but less frequent series of the seizure bursts was found (*α*_*1*_ region in Fig. 4f) and more randomly distributed seizure bursts were followed after the maximal seizure activities, which potentially represents epileptogenesis in this Dravet syndrome model^43^.

In conclusion, we presented an integrated zebrafish analysis platform, iZAP for long-term high-throughput electrophysiological monitoring. Due to the user-friendly operation and the non-invasive electrical readout on non-immobilized zebrafish, our electrographic monitoring could achieve almost weeklong continuous EEG recording of multiple zebrafish larvae. As proof-of-concept demonstration, our iZAP successfully captured monitored electrographic seizures events in two different models. We also demonstrated anti-epileptic testing in an efficient cross-over design manner using two drugs used clinically in DS patients^30^. The integrated electrophysiological microfluidic system will greatly facilitate electrophysiological monitoring on zebrafish models in the fields of bioscience and pharmaceutical applications.

## Online Methods

### iZAP system

The microfluidic unit is prepared as polydimethylsiloxane (PDMS) replicates from a SU8 photoresist mold. The SU8 mold is fabricated as gray-scale photolithography using an optical diffuser. First, a chromium mask negatively patterned for the fluidic pattern was fabricated using electron evaporation (Rocky Mountain Vacuum Tech, Inc.) and lift-off process using life-off photoresist (LOR 3A, Microchem Corp.) Then, a thick SU8 layer (approximately 1 mm thickness) was poured on the chromium-patterned side and soft-baked at 95 °C for 20 hr. The SU8-coated wafer was exposure with UV of 450 mJ·cm^−2^ on a mask aligner (OAI Series 200 Aligner) through an Opal diffuser (Edmund Optics Inc.). After 95 °C post exposure bake for 1 hr, a long-term development was applied in SU8 developer (Microchem Corp.) until a full development which commonly took 10 to 12 hr. After rinsing IPA, the SU8 mold was dried in a dark room overnight and silanized with tridecafluoro-1,1,2,2-tetrahydrooctyl trichlorosilane in a vacuum chamber for 3 hr. PDMS (Sylgard 184, Dow Corning) was poured on the SU8 mold and cured at room temperature for 1 day.

ITO-coated PET film (60 Ω per sq, Sigma-Aldrich) is used as a substrate for patterning the surface electrodes. First, 2 nm titanium and 10 nm platinum were deposited using the electron-beam evaporator on PET film patterned with LOR and SU8. Then, the contact electrode patterns were attained after life-off using 80 °C heated PG remover (Microchemicals). In the following fabrication of conductive ITO track, S1818 positive photoresist (Microchem Corp.) was patterned to protect ITO from HCl etching. After ITO etching, S1818 layer was removed using AZ 400 K (Microchem Corp.) and 5 μm-thick SU8 layer was patterned as a passivation layer with openings on the contact electrodes. Finally, the substrate was micromachined using a CO_2_ laser cutter (Versa Laser) to make two openings matching to the inlet and outlet chambers and bonded with the fluidic unit.

A home-built circuit board integrated with contact springs (Mill-Max Manufacturing Corp.) and 32-channel amplifier boards (Intan Technologies, LLC.) were used an electronic interface with a plastic housing. For a home-built Faraday cage, we made a big cutout (7 cm × 9 cm) for ITO/PET film window and several 1-mm holes for electrical wires on an aluminum enclosure (18.5 cm × 11.5 cm × 3.5 cm). Simultaneous video monitoring was obtained at 30 Hz and could fail to detect very fast tail movements, such as those associated with a C-bend escape response. Fast tail movements, in non-paralyzed zebrafish recordings, could also contribute to some false positive identifications using our seizure detection software; these false positives are minimized by individual visual analysis of the EEG records or in recordings using α-bungarotoxin or pancuronium (Figs S3 and S6).

### Zebrafish and drugs

Scn1Lab zebrafish embryos were a kind gift from Herwig Baier. Adult HuC:GFP zebrafish were a kind gift from Stephen Ekker. Zebrafish care and maintenance were performed in accordance with the guidelines of Institutional Animal Care and Use Committee at University of California at San Francisco. All experimental protocols were approved by Institutional Animal Care and Use Committee at University of California at San Francisco (AN108659-02). Zebrafish larvae were maintained in embryo medium consisting of 0.03% Instant Ocean (Aquarium Systems, Inc., Mentor, OH, USA) in deionized water containing 0.002% Methylene Blue as a fungicide. Larval zebrafish clutches were bred from scn1Lab heterozygous animals Pentylenetetrazole, valproic acid, topiramate, α-Bungarotoxin and pancuronium bromide were purchased from Sigma-Aldrich and dissolved in the embryo media before treatment.

## Additional Information

**How to cite this article**: Hong, S.G. *et al*. A Novel Long-term, Multi-Channel and Non-invasive Electrophysiology Platform for Zebrafish. *Sci. Rep*. **6**, 28248; doi: 10.1038/srep28248 (2016).

## Supplementary Material

Supplementary Information

Supplementary Video S1

Supplementary Video S2
