# Supplementary material for: A Novel Long-term, Multi-Channel and Non-invasive Electrophysiology Platform for Zebrafish
Source: Sci Rep. 2016 Jun 16;6:28248. doi: 10.1038/srep28248 (PMC4910293; doi:10.1038/srep28248)

**Supplementary Information for**

**A Novel Long-term, Multi-Channel and Non-invasive
Electrophysiology Platform for Zebrafish**

**SoonGweon Hong^12^, Philip Lee^1^, Scott C. Baraban^34*^ & Luke P. Lee^1256*^**

**Figure S1.** Fabrication and assembly of the fluidic unit and the surface electrodes

**Figure S2.** Electrographic comparison between iZAP and tissue-inserted microelectrodes.

**Figure S3.** Electrographic monitoring of iZAP on a zebrafish larva by α-Bungarotoxin and PTZ treatment.

**Figure S4.** Analysis of linearity and seizure-burst interval from the long-term monitoring of *scn1Lab* mutant (Fig. 4F).

**Figure S5.** Drug screening demonstration of TOP and VAP crossover test.

**Figure S6.** Description of the seizure score algorithm.

**Figure S7.** Electrographic monitoring of iZAP on a wild-type (TL) zebrafish larva by pancuronium bromide and PTZ treatment.

**Figure S8.** Seizure-like electrographs of *scn1Lab* mutant with and without paralyzing agent treatment and an age-match control.

**Movie S1.** Intact locomotion of zebrafish larvae retrieved after 130-hour monitoring

**Movie S2.** Time-lapse electrographic signals of the PTZ-induced acute model.

**Figure S1.** Fabrication and assembly of the fluidic unit, the surface electrodes and the electronic unit. **(A)** Steps of surface electrode fabrication: (i) Platinum was deposited for the contact electrodes on top of ITO coated PET film through LOR/SU8 photolithography. (ii) ITO was patterned using S1818 etching mask and HCl etching. (iii) SU8 passivation layer was patterned as 10-µm thick. (iv) Using CO2 laser, two openings was made for zebrafish loading and media outlet. **(B)** Steps for microfluidic unit fabrication: (i) 150-nm thick chromium layer was patterned on a glass wafer. (ii) 1-mm thick SU8 layer was prepared up to softbaking. (iii) Using an opal diffuser, the thick SU8 layer was patterend for the gray-scale structure. (iv) The PDMS microfluidic device was replicated from the SU8 mold. **(C)** Layout of integtrated microfluidic device with the surface electrode substrate. **(D)** Assembly of the three components. **(E)** A cross-section view of zebrafish trapping inside the integrated microfliudic unit. *l_channe_*_l_ is longer than *l_larva_* but shorter than *2·l_larva_* .


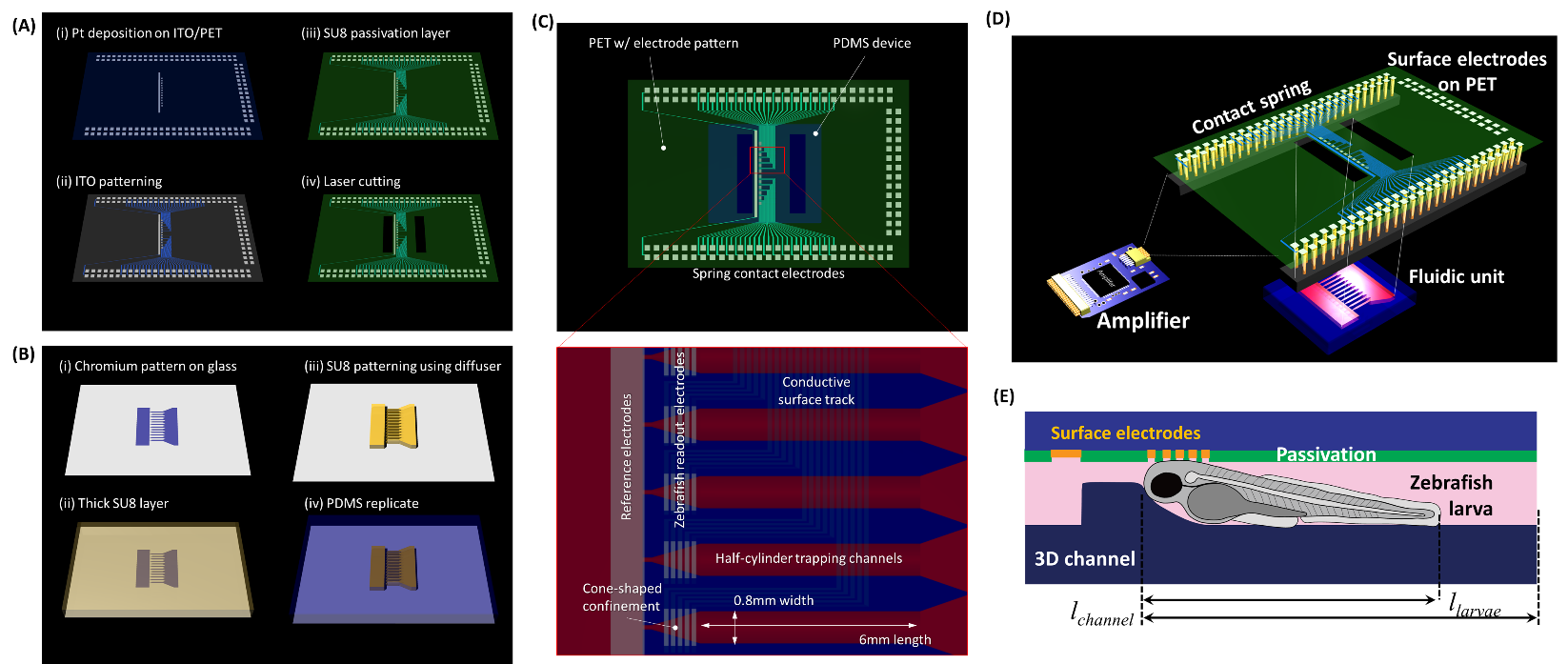


**Figure S2.** EEG comparison between measurements of local field potential using penetrating a microelectrode and surface electrodes in iZAP. Red lines in the zebrafish cartoon indicate the electrodes.


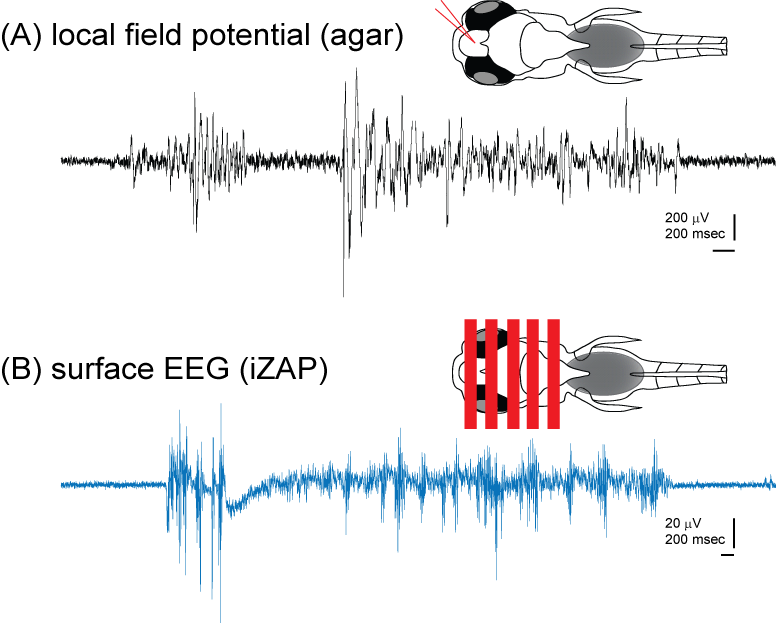


**Figure S3.** EEG measurement in iZAP with α-Bungarotoxin (paralyzing agent; 20-min pretreatment) and PTZ treatment. The significant reduction of zebrafish motions (i.e., tail and fin movement) was attained through α-Bungarotoxin treatment, and a series of seizure-like bursts were observed under PTZ treatment, identical to the α-Bungarotoxin non-treated cases.


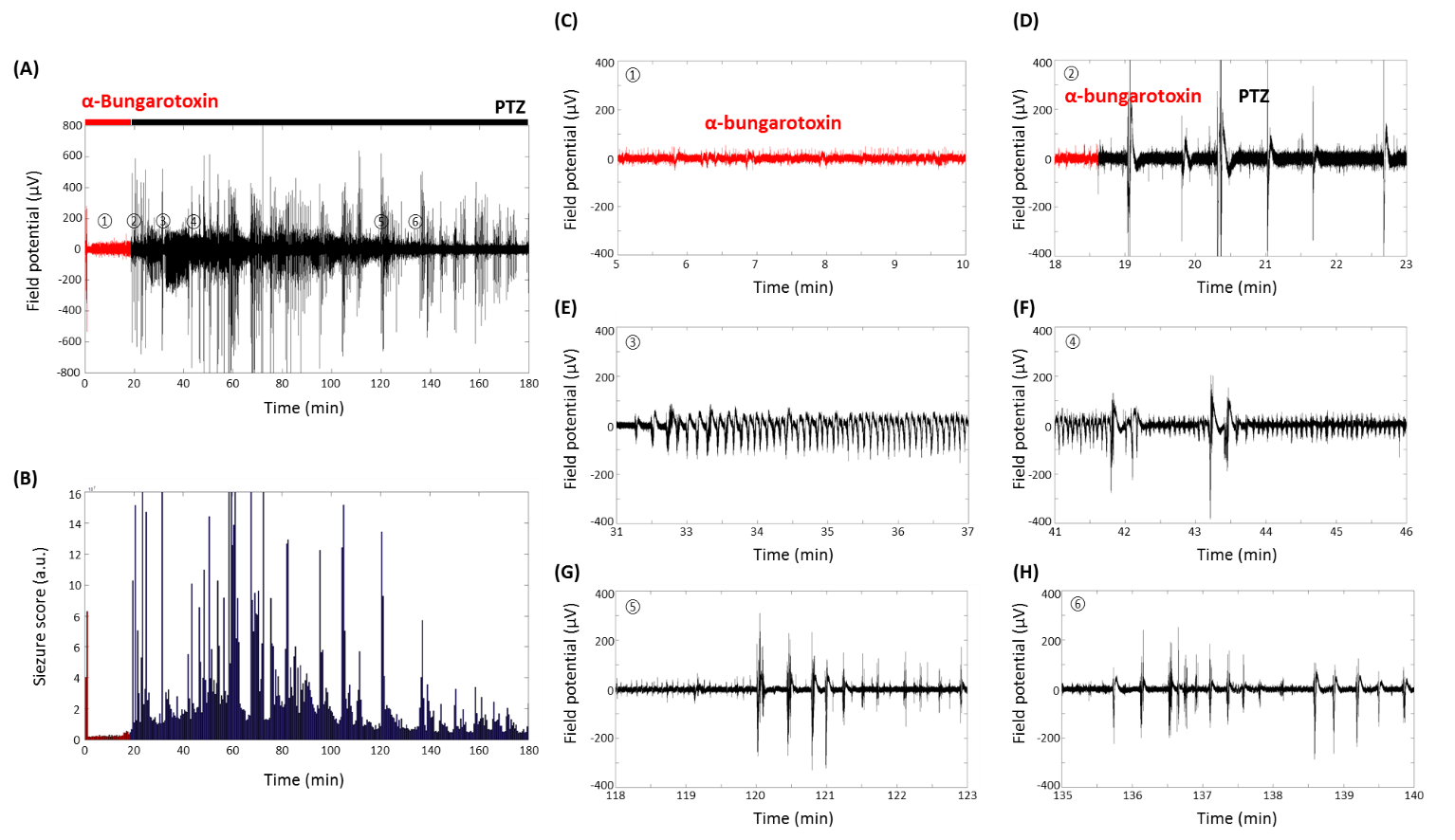


**Figure S4.** Linearity analysis of the cumulative frequency of seizure events shown in Fig. 4F. Three regions were shown to be linear-like with high values of linear regression (R-squared) as indicated in (A), (C) an (E). The intervals between seizure-like bursts were characterized as frequency histograms (B, D and F) and showed Poisson-like distribution in the region of higher linearity. Around the period of maximal seizure activity (26-28hr), the seizure-like events happened frequently as 35-40sec interval.


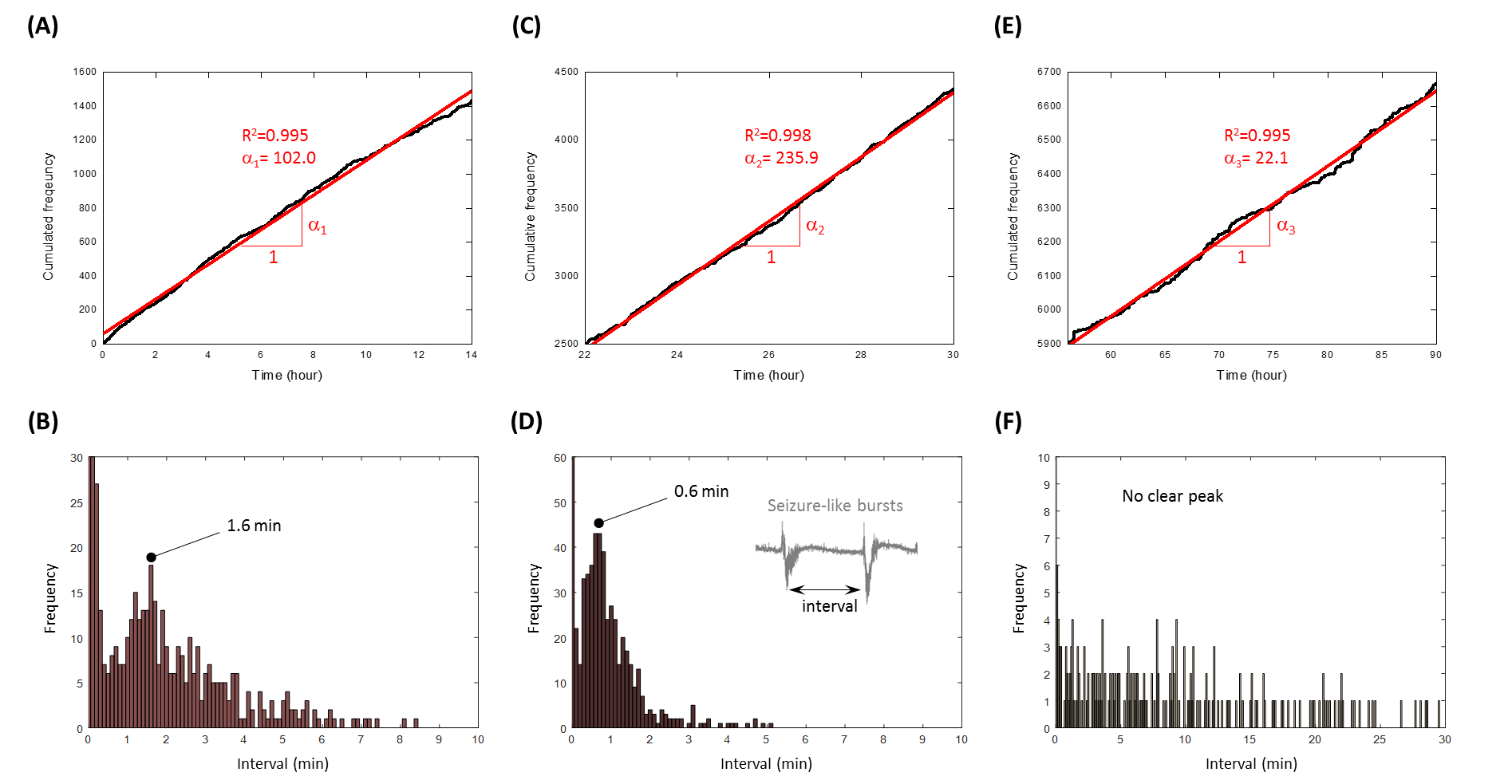


**Figure S5.** Drug screening demonstration of iZAP system as paired with Fig. 5 in the main text. **(A, B)** A representative EEG signals and corresponding 5-min interval seizure score bar plot for TOP and VPA crossover test. **(C, D, E, F, G)** Electrograph and corresponding seizure score plot for each stage of the crossover test. **(H)** Drug efficacy plot based on total seizure score from 9 *scn1Lab* mutants as baseline-normalized.


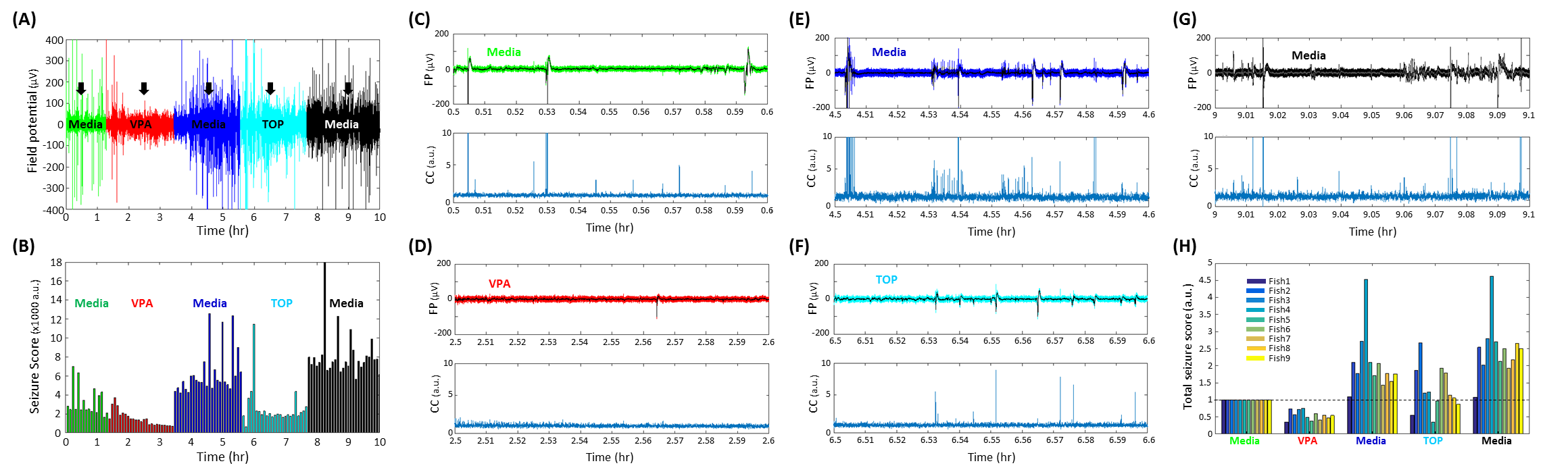


**Figure S6.** Description of the seizure scoring algorithm permitting to discriminate motion artifacts. The software algorithm follows time derivative of recorded field potentials, cross-correlation among a set of time derivatives assigned to a single trapping channel, and time integration of the cross-correlation over a long-term period (i.e., 30 sec in this figure). The step of time derivative screens out field potentials with slow changes in time; the step of cross-correlation highly score synchronous electrical activities in space; the step of time integration highlights long-term electrical activities over temporally brief motion-associated artifacts. **(A, B and C)** Each characterization plot for seizure-like episode induced by PTZ. **(D, E and F)** Each characterization plot for baseline of the wild-type strain (TL). In this comparison, the time integration resulted in more than 20 times higher seizure score in the seizure-like episode.


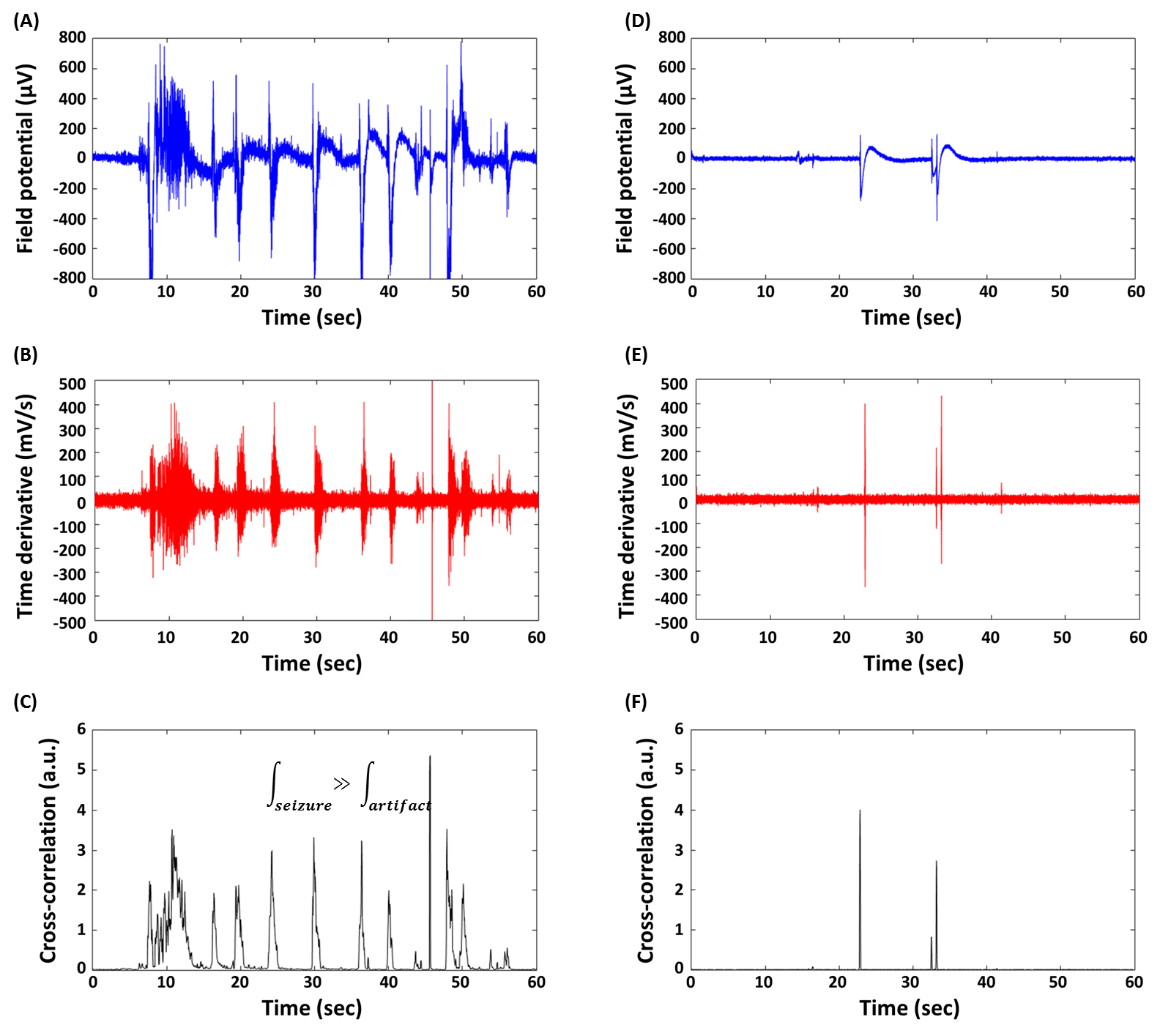


**Figure S7.** EEG measurement in iZAP with pancuronium bromide (paralyzing agent) and PTZ treatment on the wild-type TL strain. The significant reduction of zebrafish motions (i.e., tail and fin movement) was attained through pancuronium treatment, and a series of seizure-like bursts were observed during the successive PTZ treatment while the locomotion activities were eliminated. **(A)** 4-hour filed potential plot for successive treatment of pancuronium and PTZ treatment. **(B and C)** Zoomed field potential in (A).


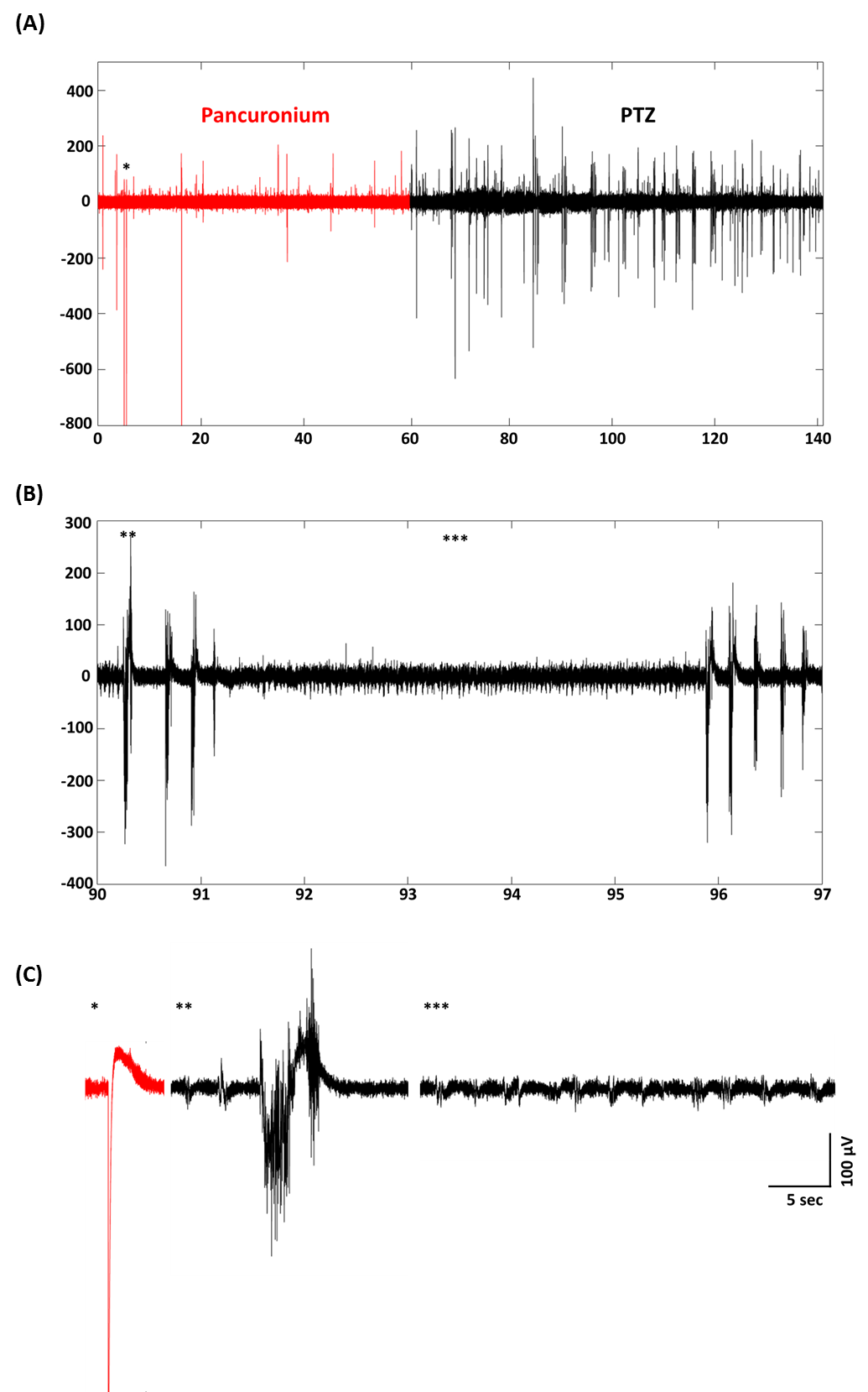


**Figure S8.** Electrophysiological monitoring on the *scn1Lab* mutant and an age-matched control of 5 dpf with pancuronium bromide treatment. Whereas the *scn1Lab* mutant showed seizure-like electrical bursts in both of normal embryonic media (baseline) and pancuronium bromide, the age-matched control showed motion-associated artifacts in the baseline recording and decreased artifacts in the pancuronium bromide treatment.


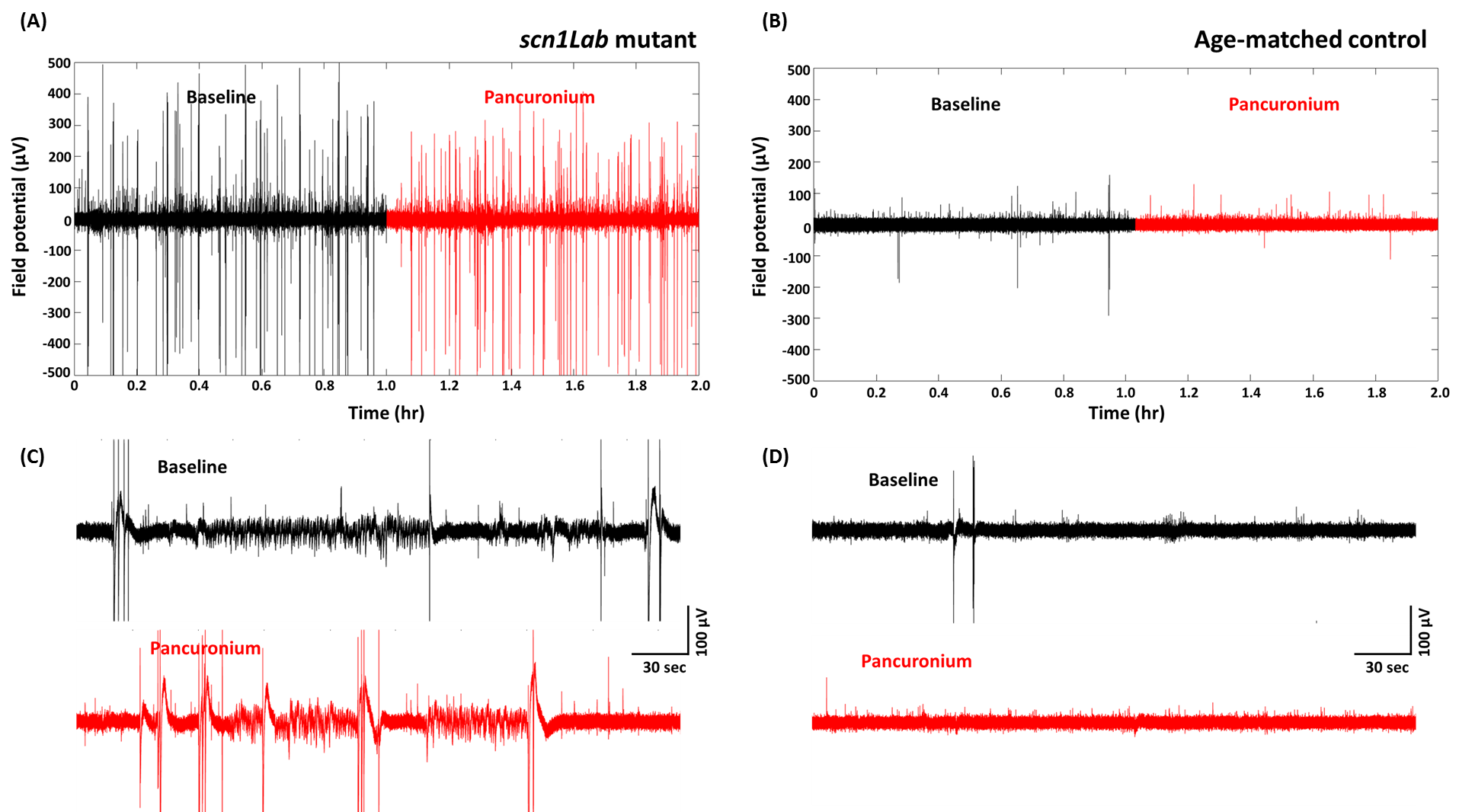

Supplement: Supplementary Information [file srep28248-s1.doc]
